# Wired for motherhood: induction of maternal care but not maternal aggression in virgin female CD1 mice

**DOI:** 10.3389/fnbeh.2015.00197

**Published:** 2015-07-23

**Authors:** Ana Martín-Sánchez, Guillermo Valera-Marín, Adoración Hernández-Martínez, Enrique Lanuza, Fernando Martínez-García, Carmen Agustín-Pavón

**Affiliations:** ^1^Laboratory of Functional Neuroanatomy (NeuroFun), Unitat Predepartamental de Medicina, Facultat de Ciències de la Salut, Universitat Jaume ICastelló de la Plana, Spain; ^2^Departaments de Biologia Cel·lular i de Biologia Funcional, Facultat de Ciències Biològiques, Universitat de ValènciaValència, Spain

**Keywords:** aggression, communal nesting, maternal care, maternal sensitization, outbred strain

## Abstract

Virgin adult female mice display nearly spontaneous maternal care towards foster pups after a short period of sensitization. This indicates that maternal care is triggered by sensory stimulation provided by the pups and that its onset is largely independent on the physiological events related to gestation, parturition and lactation. Conversely, the factors influencing maternal aggression are poorly understood. In this study, we sought to characterize two models of maternal sensitization in the outbred CD1 strain. To do so, a group of virgin females (godmothers) were exposed to continuous cohabitation with a lactating dam and their pups from the moment of parturition, whereas a second group (pup-sensitized females), were exposed 2 h daily to foster pups. Both groups were tested for maternal behavior on postnatal days 2–4. Godmothers expressed full maternal care from the first test. Also, they expressed higher levels of crouching than dams. Pup-sensitized females differed from dams in all measures of pup-directed behavior in the first test, and expressed full maternal care after two sessions of contact with pups. However, both protocols failed to induce maternal aggression toward a male intruder after full onset of pup-directed maternal behavior, even in the presence of pups. Our study confirms that adult female mice need a short sensitization period before the onset of maternal care. Further, it shows that pup-oriented and non-pup-oriented components of maternal behavior are under different physiological control. We conclude that the godmother model might be useful to study the physiological and neural bases of the maternal behavior repertoire.

## Introduction

In mammalian species, maternal care has a deep impact in the development of newborns. The quality and the quantity of this behavior influence some phenotypical aspects of the offspring (Meaney, 2001; Pedersen et al., 2011; Pan et al., 2014), such as stress reactivity (Francis et al., 1999; Meaney, 2001; Champagne et al., 2003; Suomi, 2006) and alterations in neuroendocrine regulation. Among other factors, there is a substantial upregulation of hippocampal glucocorticoid receptors and hypothalamic corticotropin-releasing factor, along with stress-related hormone levels (Meaney, 2001; Vaiserman, 2015). All these factors contribute to increased vulnerability for some affective disorders later in life (Larsen and Grattan, 2012; Zhang et al., 2013). In fact, low maternal care at infancy is associated with significantly increased risk of child neglect/abuse and depression during adulthood (Canetti et al., 1997; Repetti et al., 2002; Numan and Insel, 2003; Andersen et al., 2008).

These findings evidence the importance of maternal care as a regulator of a healthy development of the infants. Thus, investigating the mechanisms of mother-infant interaction is essential for understanding how to improve bonding and promote physical and mental health for future generations. To this aim, we need animal models in which to study the neural substrate of maternal behavior.

The rat is the species of choice in most of the studies on the neurobiological underpinning of maternal behavior. In rodents, similarly to other mammals, the maternal behavior repertoire includes pup-directed—retrieval and grouping pups in nest, crouching, pup-licking/grooming-, and non-directed responses such as nest building and maintenance and maternal aggression, mainly directed to defend the nest (Vom Saal et al., 1995).

The rat model has provided a wealth of data on the neurobiological and physiological mechanisms of pup-directed behaviors, including the mechanisms of induction of maternal care in virgin females. Thus, when virgin adult rats are exposed to pups for the first time, they first avoid them (Rosenblatt, 1967; Fleming and Rosenblatt, 1974), then tolerate pups after 2–3 days of exposure, and finally display maternal-like care after 5–7 days (Rosenblatt, 1967; Fleming and Luebke, 1981). This so-called maternal sensitization apparently depends on hormonal factors, since blood transfusions from a parturient female into virgin female rats (Terkel and Rosenblatt, 1972) facilitate the onset of maternal behavior. Hormonal treatments with physiological levels of progesterone and estradiol in virgin females (Bridges, 1984; Stern and McDonald, 1989; Bridges et al., 1990) in conjunction with prolactin seem to shorten the sensitization period, thus confirming that maternal care is controlled by endocrine factors acting around parturition.

In contrast to rats, some primate virgin females display spontaneous maternal behavior (Maestripieri and Wallen, 1995). Similarly, adult virgin female laboratory mice show near-immediate maternal care (Calamandrei and Keverne, 1994; Stolzenberg and Rissman, 2011; Alsina-Llanes et al., 2015). In this context, investigations on the neuroendocrinology of maternal behavior in mice might be relevant to understand the same aspects in primates, including humans.

The induction of maternal care purely by contact with newborns in mice and other species (Olazábal and Young, 2006) –without the need of the physiological changes occurring during pregnancy and lactation–, suggests that there might be a wired mechanism in some mammalian females triggering maternal care spontaneously in response to pup stimuli. However, studies on maternal sensitization have not addressed the induction of maternal-like aggression by continuous exposure to pups (Fleming and Luebke, 1981; Stolzenberg and Rissman, 2011; Alsina-Llanes et al., 2015). Investigating whether pup-induced sensitization results in an aggressive state in virgin females might help understand the neural basis of maternal aggression, a conserved behavior in most mammalian species, including humans (Hahn-Holbrook et al., 2011).

In the present study, we sought to characterize two different protocols of maternal sensitization in which to study both aspects of maternal behavior in mice i.e., maternal care and maternal aggression. The strain contributes to variability in maternal behavior in mice (Parmigiani et al., 1999; Numan and Insel, 2003; van der Veen et al., 2008, but see Gandelman et al., 1970), and inbred strains usually display lower maternal care measures than outbred ones. Thus, we investigated whether our sensitization procedures were capable of inducing both pup-directed responses and maternal aggression in the outbred CD1 strain.

## Materials and Methods

### Subjects

For the present study, we used 100 experimental adult females (9–14 weeks of age) of Swiss albino CD1 strain (Janvier Labs, Le Genest-Saint-Isle, Saint-Berthevin Cedex, France). In addition, we employed 12 stud males and 23 adult intact males and 15 castrated males unrelated to the females for maternal aggression tests (Janvier Labs, Le Genest-Saint-Isle, Saint-Berthevin Cedex, France). All animals were housed in polypropylene plastic cages with *ad libitum* access to water and food (Teklad Global 14% Protein Rodent Maintenance Diet, Harlan). During pregnancy and testing animals were housed in black propylene plastic cages (145 mm wide, 465 mm length, and 215 mm high).

The room was maintained at 24°C, 60–80% relative humidity and a 12:12 h light:dark cycle, with lights on at 08:00 h. Cages were cleaned weekly, except during postpartum days, when dams were left undisturbed until the end of the experiment.

Animals were treated according to the EEC guidelines of June 3, 2010 (6106/1/10 REV1), and all procedures were approved by the Committee of Ethics on Animal Experimentation of the University of Valencia.

### Experiment 1. Pup-Directed Maternal Behavior in Dams, Godmothers and Pup-Sensitized Females

Adult virgin females were randomly assigned to three groups: experimental dams (*n* = 7), experimental godmothers, i.e., virgin females sharing pup-care with dams since the moment of parturition (previously defined by Martín-Sánchez et al., 2015; *n* = 7) and pup-sensitized females (*n* = 8), i.e., virgin pup-naïve females that were exposed after the first test to 2 h daily sessions of sensitization. The most typical laboratory condition for studying maternal behavior in rodents is the housing of a mother alone with her litter. However, in our experimental design we aimed to compare godmothers with dams. Since godmothers are necessarily housed with a dam, all groups, including dams, were housed in pairs to allow for a direct comparison of their behavior. Females assigned to be dams were paired with a stud male (*n* = 4) for 4 days. After mating, males were removed and pregnant females were housed in pairs with an accompanying female.

**Figure 1:**
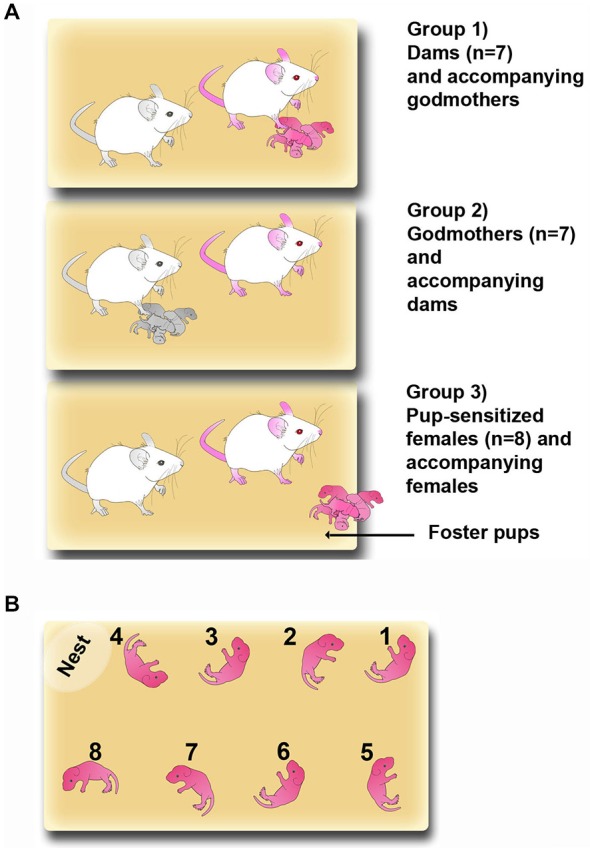
**Maternal sensitization models. (A)** Sketch of the three experimental groups: Group (1) dams and accompanying godmothers; Group (2) godmothers and accompanying dams; and Group (3) pup-sensitized females and accompanying virgin females. Pup-sensitized females were isolated and exposed to pups 2 h daily. Pink-colored drawings represent the experimental females and black and white drawings are the accompanying females. **(B)** Sketch showing the detailed procedure for placing pups in the pup-retrieval test.

The experimental groups are shown in Figure 1A. In the group 1, dams, and group 2, godmothers, pregnant dams were housed with a sister (the godmother). They remained together during the rest of gestation, parturition and lactation. The day of birth was considered as postpartum day 0 (PPD 0). On PPD 1, litters were culled to eight pups following Martín-Sánchez et al. (2015). Thus, group 1 dams and group 2 godmothers (Figure 1A, colored females) were the experimental animals, whereas group 1 godmothers and in group 2 dams acted as accompanying females (Figure 1A, black and white females). Finally, for the group 3, pup-sensitized females, two virgin females were housed together. One of the females of each pair was sensitized for 2 h per day exposition to foster pups throughout the experimental phase (see below).

Previous observations in rats indicated that a short separation (less than 1 h) of dams from pups resulted in a dramatic increase of maternal care (Pryce et al., 2001). To avoid this confounding effect, the accompanying female of each group was not tested.

Pup-directed maternal behavior tests were performed daily in the females’ home cages from PPD 2–4 between 09:00–14:00 h. Females were brought to the testing room in their home cage immediately before the test. Since females were housed in pairs (dam/godmother in groups 1 and 2, two virgin females in group 3), the accompanying female was removed prior to the test and put in an adjacent clean cage.

In the groups of dams and godmothers, the eight pups were briefly removed with a clean spatula. Pup-sensitized females received eight stimulus pups from a donor dam. The experimenter scattered the pups with a clean spatula along the whole perimeter of the cage, approximately in the same location for all the females (Figure 1B). Testing lasted 40 min and behaviors were video-recorded for their following analysis. Behavioral measures are given below.

After the test, the accompanying females of experimental dams and godmothers were returned to the home cage. Pup-sensitized females were left undisturbed with the pups for 2 h. After this exposure period, pups were returned to their home cage with the donor dams, and pup-sensitized females reunited with their cage mates. The full procedure was repeated for three consecutive days.

### Experiment 2. Maternal Aggression in Dams, Godmothers, Pup-Sensitized Females and Pup-Naïve Females

We next checked whether the different protocols that induce pup-directed maternal behavior could also elicit maternal aggression. Thus, we performed a maternal aggression test using four experimental groups: dams (*n* = 9), godmothers (*n* = 9), pup-sensitized females (*n* = 9) and pup-naïve females (*n* = 9). All dams were housed together with godmothers, and pup-sensitized females were grouped with pup-naïve ones, as in Experiment 1. Pup-sensitized females were exposed 2 h daily to eight foster pups for 4 days. On PPD5, we performed a maternal aggression test, using males as intruders (*n* = 15), following Martín-Sánchez et al. (2015). Each male was used in no more than three nonconsecutive tests, and the interval between tests was at least 1 h. To avoid damage to the males and a possible effect of experience, a male was used only once as an intruder for dams.

Maternal aggression tests were performed in the females’ home cages between 09:00–14:00 h on PPD 5. We selected this time point because it is when maternal aggression shows the highest expression level, after which it declines during the second postpartum week (Gandelman, 1972; Lonstein and Gammie, 2002). Females were brought to the testing room in their home cage. Pups were removed prior to the tests (Svare et al., 1981; Lonstein and Gammie, 2002), to avoid any infanticide behavior by the male intruder (Vom Saal and Howard, 1982). During the test, pups and accompanying females were left in separate adjacent cages. For the aggression test, an unrelated, adult male intruder was placed in the female’s cage for 5 min. Intruders were different from the stud males. The procedure was identical for all virgin females.

### Experiment 3. Maternal-Like Aggression and Interaction with Males in Godmothers in the Presence and the Absence of Pups

In order to evaluate whether godmothers (*n* = 6) would display aggressive behaviors in the presence of pups to defend, we performed a resident-intruder test in absence and presence of pups. In this test, we also analyzed the interaction of godmothers with males, i.e., general sniffing and anogenital approaches. These tests were performed following the same protocol as in Experiment 2. On PPD4, dams and pups were removed from the cage and an unrelated male (*n* = 6) was put into the arena. On PPD5, the dam was removed and the resident-intruder test was done in the presence of the pups. In the event of any infanticide behavior, the test was finished immediately by removing the intruder. The accompanying dams (*n* = 6) were tested immediately after the godmothers in the absence of pups.

### Experiment 4. Maternal Aggression as a Territorial Behavior

To evaluate whether the display of maternal aggression reflects a territorial behavior or an increased drive to attack unknown conspecifics, we compared the response to a male conspecific of dams and godmothers in a neutral arena (*n* = 10 per group). To minimize the proactive approaches of males to the females, we used castrated males that were swabbed in both neck and anogenital region with 5 μl of intact male urine just before the test, a protocol that elicits the same levels of maternal aggression when tested at the home cage as the use of an intact male (Martín-Sánchez et al., 2015).

All tests were performed at PPD5. Each female was individually placed in a 220 × 220 × 145 h mm cage. After a habituation period of 2 min, we placed the intruder into the arena and recorded the behavioral responses of the females during 5 min.

### Behavioral Measures

#### Pup-Directed Maternal Behavior

We scored the latency to sniff the first pup and to retrieve the first three pups during the first 300 s of the 40 min tests. If a female did not retrieve them, it was assigned a latency value of 300 s. Fifteen minutes after starting the test, the observer scored other pup-directed maternal behaviors for 10 min (between minute 15 and 25) following Pryce et al. (2001). Specifically, we scored the frequency of crouching defined as an immobile posture with all four limbs supported, acquiring a slightly arched position over the pups, so that pups had access to the female’s ventral surface. In addition, we scored pup licking/grooming, defined as an active behavior in which the female caught a pup with both forelimbs and then approached a pup to the mouth/nose. To measure the frequency of crouch and pup licking/grooming we adapted the protocol by Stolzenberg and Rissman (2011). We observed the mice every 15 s during the 10 min period of observation (40 total time slots), and counted a positive event if the females were expressing crouching and/or pup licking/grooming behaviors at the time of each observation.

Additionally, we scored the number of events that a female tried to carry a pup with the mouth to nest site unsuccessfully as failure in pup retrieval.

#### Risk-Assessment Behavior

In order to evaluate whether virgin mice show a neophobic response towards pups—similar to that expressed by virgin rats during the sensitization period (Fleming and Luebke, 1981)—we scored the frequency of risk-assessment behavior in the three experimental groups. We measured this behavior because mice show it as an innate response when they are confronted toward fear-evoking stimuli. We scored risk-assessment behavior following Papes et al. (2010) criteria, as a stereotypical cautious investigative approach characterized by a low-lying extended body posture.

#### Aggressive Behavior

We scored the attacks towards the intruder during the 5 min test. An attack was defined as a female spontaneously and actively biting or kicking the intruder, since the speed of the females behavior made difficult to separate these responses (Martín-Sánchez et al., 2015). We did not include as attacks the refusals—aggressive responses of the females to a male approach, consisting of kicks using any of four limbs.

#### Sniffing

To evaluate social interaction we scored total time that dams and godmothers spent sniffing any part of the body of the intruder.

#### Anogenital Investigation

To evaluate sexual-like approaches of dams and godmothers to the males, we scored the number of times that a female approached the anogenital zone of the intruder.

All behaviors were scored by an observer blind the experimental conditions using the event recorder of the video-track software SMART 2.5 (Panlab S.L., Barcelona, Spain).

### Statistical Analysis

Statistical analyses were performed using IBM SPSS Statistics 19.0. We first checked whether the data fulfilled the conditions of ANOVA: normality (Kolmogorov–Smirnov’s test), homoscedasticity (Levene’s test) and sphericity (Mauchly’s test) of the data. When normality was violated, data was either log-transformed (log[X + 1]) or we performed non-parametric analyses. Data from Experiment 1 were analyzed using ANOVA of repeated measurements with TEST (Day 1, Day 2 and Day 3) as intra-subject variable and GROUP (Dams, Godmothers and Pup-sensitized females) as inter-subject variable, or Kruskal-Wallis test. Data from Experiment 2 were analyzed using non-parametric Kruskal-Wallis test, followed by a Dunn’s *post hoc*. Data from Experiment 3 were analyzed using the non-parametric Wilcoxon test for related samples and Mann-Whitney test for non-related samples. Data from Experiment 4 were analyzed by means of Mann-Whitney tests. Significance was set at *p* < 0.05.

## Results

### Experiment 1. Godmothers and Pup-Sensitized Females Express Maternal Care with Different Time Course

#### Latency to Sniff the First Pup

In order to check whether non-lactating females avoided pups, we measured the latency to approach and sniff pups. The ANOVA of log(X + 1) transformed data showed neither significant effects of TEST (*F*_2,38_ = 0.59, *p* > 0.1) and GROUP (*F*_2,19_ = 2.959; *p* > 0.05), nor interaction between the factors TEST × GROUP (*F*_4,38_ = 0.588, *p* > 0.1). Thus, latency to sniff the first pup was similar in all the females, irrespective of their status (data not shown).

#### Risk-Assessment Behavior

To assess the possible anxiogenic properties of pups for non-lactating females we scored risk-assessment behavior in dams, godmothers and pup-sensitized virgin females. We analyzed differences between groups using a Kruskal-Wallis test. Dams, godmothers and pup-sensitized females displayed very low rate of risk assessment or avoidance responses toward pups. The analysis revealed that there were no significant differences between groups across test days (Day 1: *χ*^2^ = 4.5, Day 2: *χ*^2^ = 3.852 and Day 3: *χ*^2^ = 0.095; *p* > 0.1 in all cases; data not shown). These data suggest that virgin females with no previous experience are not neophobic toward pups.

#### Time Difference Between Sniffing and Retrieving the First Pup

**Figure 2:**
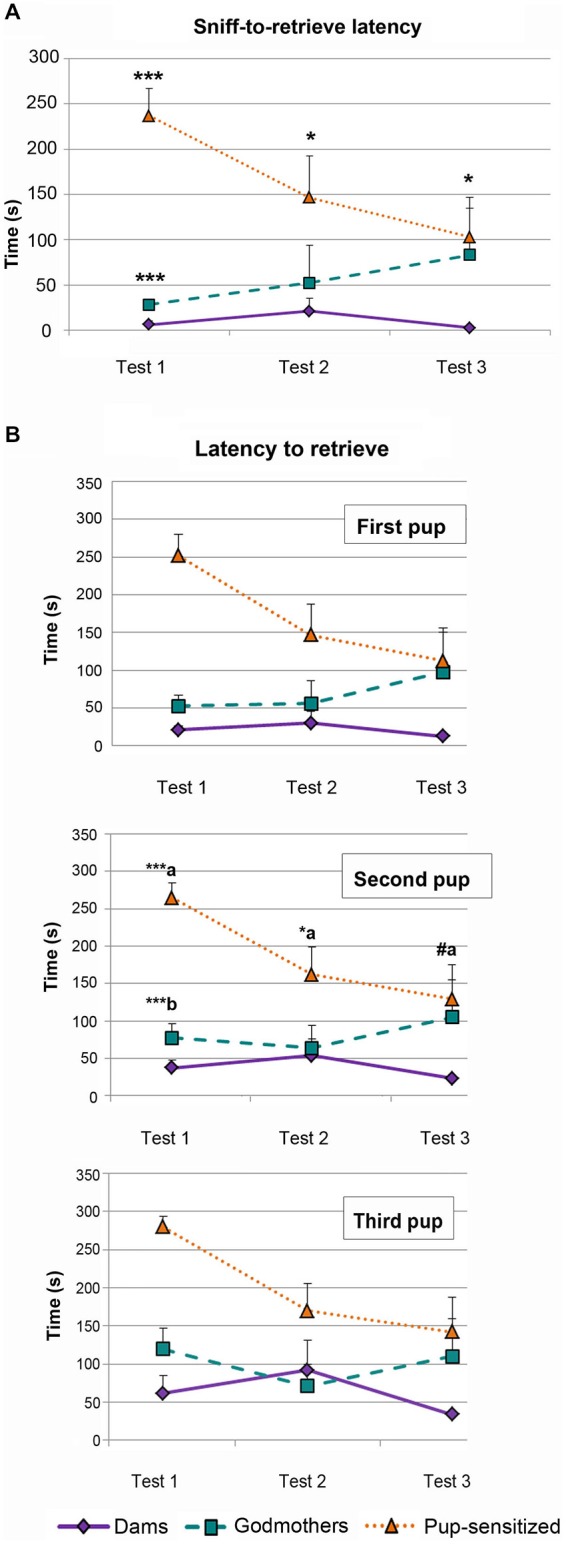
**Behavioral measures of the pup retrieval test. (A)** Time difference between first sniff and retrieval the first pup. Time difference spent by dams, godmothers, and pup-sensitized females between the first sniffing of a pup and the first pup retrieval in each test day. ANOVA of repeated measurements indicated that dams initiated faster pup retrieval after sniffing the first pup than pup-sensitized females across the 3 days of testing. Godmothers were slower than dams only on the first day. Data are represented as mean ± SEM. **(B)** Latency to retrieve the first three pups. Time that dams, godmothers, and pup-sensitized females took to retrieve the first, second and third pup. ANOVA of repeated measurements indicates that pup-sensitized females were slower to retrieve the second pup than dams on first and second days test. Pup-sensitized females retrieved the second pup slower than godmothers only on the first day. (a) comparison pup-sensitized vs. dams; (b) comparison godmothers vs. pup-sensitized. ^#^*p* = 0.06, *p < 0.05, ***p < 0.001.

The ANOVA of log(X + 1) transformed data revealed a significant effect of the factors TEST (*F*_2,38_ = 4.746, *p* = 0.014), and GROUP (*F*_2,19_ = 15.167, *p* < 0.001), as well as a significant interaction TEST × GROUP (*F*_4,38_ = 2.707, *p* = 0.044). This interaction was further explored by analyzing the simple effect of GROUP within each TEST. *Post hoc* pairwise comparisons indicate that there are differences among females in all 3 days (Day 1, *F*_1,19_ = 57.917, *p* < 0.001; Day 2, *F*_1,19_ = 4.915, *p* = 0.019; Day 3, *F*_1,19_ = 5.539, *p* = 0.013). Pairwise comparisons among the factor GROUP showed global statistically significant differences between dams and pup-sensitized females (*p* < 0.001) and godmothers and pup-sensitized females (*p* = 0.02; Figure 2A). *Post hoc* analysis with Bonferroni’s correction revealed that both godmothers (*p* < 0.001) and pup-sensitized females (*p* < 0.001) took more time to retrieve the first pup after the first sniff as compared to dams on Day 1. The analysis also showed significant differences between godmothers and pup-sensitized females in this measure (*p* < 0.001). On the next days, only pup-sensitized females behaved different from the lactating females (Day 2, *p* = 0.03 and Day 3, *p* = 0.011), whereas godmothers were as quick as dams to retrieve the first pup after sniffing it (Figure 2).

#### Latency to Retrieve the First Three Pups

We analyzed log(X + 1) transformed data for each pup. For all the pups, the results revealed a highly significant effect of both TEST (Pup 1: *F*_2,38_ = 4.273, *p* = 0.021; Pup 2: *F*_2,38_ = 6.095; and Pup 3: *F*_2,38_ = 7, 358; *p* < 0.005) and GROUP (*F*_2,19_ = 11.445; *F*_2,19_ = 9.747, *F*_2,19_ = 7.828; *p* < 0.003 in all cases). Further analyses of these effects indicated an expected global improvement of pup retrieval through the test, rendering a reduction of the latency between Days 1 and 3, which became significant for pups 2 and 3 (*p* < 0.03). Concerning differences between groups, dams showed a significantly shorter latency to retrieve pups than pup-sensitized virgins (*p* < 0.001 for the three pups). Godmothers showed the same retrieval latency than dams (*p* > 0.12, *p* > 0.32, *p* > 0.54 for pups 1, 2 and 3 respectively), while differences between godmothers and pup-sensitized females bordered on significance (*p* = 0.06, *p* = 0.05, *p* = 0.07 for pups 1, 2 and 3 respectively).

Only data from the second pup showed significant TEST × GROUP interaction (*F*_4,38_ = 2.841; *p* < 0.05; Figure 2B, Second Pup). Further analysis of this interaction by means of a multivariate test of the effect of TEST within each GROUP indicates that whereas dams and godmothers showed a steady performance in pup retrieval through the days (*p* > 0.18 in both cases), virgin pup-sensitized females improved in their performance (*p* = 0.004). *Post hoc* pairwise comparisons with Bonferroni corrections indicate that retrieval latency significantly decreased between days 1 and 2 (*p* = 0.018) and days 1 and 3 (*p* = 0.003) in pup-sensitized females, but did not differ between days 2 and 3. The comparison of the performance of the different females within each day (Figure 2B) revealed that dams and godmothers did not differ in any of the days (*p* > 0.07), whereas pup-sensitized females showed significantly longer pup retrieval latency than dams on days 1 (*p* < 0.001) and 2 (*p* = 0.038), and reached a performance nearly similar to dams on day 3 (*p* = 0.06). Differences between godmothers and pup-sensitized virgins were restricted to day 1 (*p* < 0.001).

In conclusion, godmothers were not significantly different from dams in any respect, and should be considered fully maternal from the first testing day. By contrast, pup-sensitized females became maternal through the tests and needed one or two sensitization sessions to display fully maternal pup-retrieval behavior.

#### Frequency of Failures in Pup Retrieval

In order to check whether differences in pup retrieval between females could be attributed to differential motor performance, we analyzed the frequency of failures in pup retrieval behavior between groups using a Kruskal-Wallis test. All experimental groups displayed a low rate of failures in pup retrieval and no statistical differences in this measure were observed across tests (Day 1, *χ*^2^ = 2.637; Day 2, *χ*^2^ = 4.359; Day 3, *χ*^2^ = 1.799; *p* > 0.2 in all cases, data not shown).

#### Frequency of Crouching

**Figure 3:**
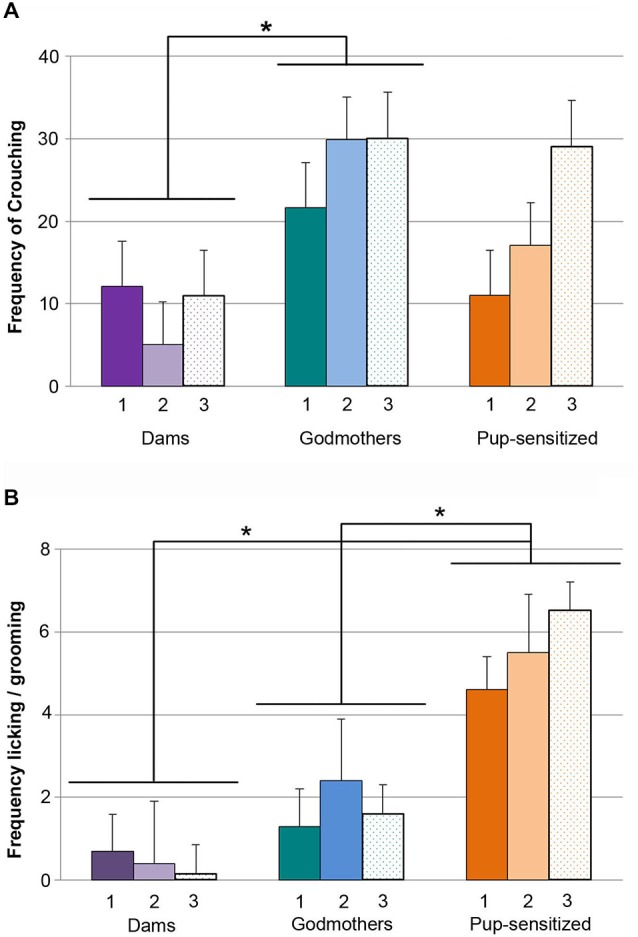
**Godmothers and pup-sensitized females differentially expressed crouching and licking/grooming.** Number of observations (out of 40 possible events) of crouching and licking/grooming during the 10 min period of observation **(A)** Godmothers displayed crouching more frequently than dams but not than pup-sensitized females. **(B)** Pup-sensitized females showed pup licking/grooming more frequently than dams and godmothers. *p < 0.05.

The ANOVA showed that there were significant differences in crouching between groups (*F*_2,19_ = 4.364, *p* = 0.028, Figure 3A), and a significant effect of TEST (*F*_2,18_ = 4.683, *p* = 0.023), but not of TEST × GROUP interaction (*F*_4,38_ = 2.239, *p* = 0.083). Further comparisons between groups showed statistically significant differences between godmothers and dams, indicating that godmothers displayed crouching more frequently than dams across test days (*p* = 0.025), but there were no differences between godmothers and pup-sensitized (*p* > 0.5) or dams and pup-sensitized females (*p* > 0.3).

#### Frequency of Pup Licking/Grooming

The analysis of log(X + 1) transformed data showed a significant effect of the variable GROUP (*F*_2,19_ = 13.553, *p* < 0.001, Figure 3B), but no effect of TEST or TEST × GROUP interaction. *Post hoc* comparisons between groups confirmed that pup-sensitized females overexpressed licking behavior as compared with both dams (*p* > 0.001) and godmothers (*p* = 0.021).

### Experiment 2. Contact with Pups Fails to Elicit Maternal Aggression in Godmothers and Pup-Sensitized Females

**Figure 4:**
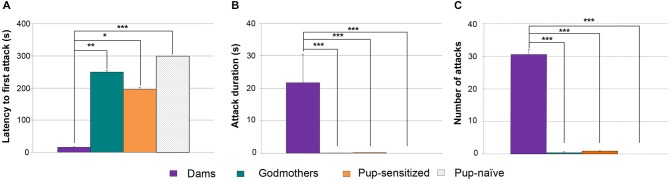
**Both maternal sensitization models failed to promote maternal-like aggression. (A)** Time that dams, godmothers, pup-sensitized and pup-naïve females took to initiate the first attack. Kruskal-Wallis test indicated that dams were faster than all the rest of groups to initiate maternal aggression toward a male intruder. Dams displayed longer attack duration **(B)** and **(C)** higher number of attacks toward male intruder in comparison with godmothers, pup-sensitized females and non-sensitized virgin females. Data are represented as mean ± SEM. *p < 0.05; **p < 0.01; ***p < 0.001.

Since both procedures were able to induce full maternal care, we wondered whether pup-sensitized virgin females would display nest defense. Therefore, we applied a Kruskal-Wallis test to evaluate differences in latency to attack, total time attack duration and frequency of attacks in dams, godmothers, pup-sensitized and pup-naïve virgin females when confronted to an adult male intruder. Data revealed global, statistically significant differences in the latency to attack (*H* = 23.2; *p* > 0.001 Figure 4A), total attack duration (*H* = 26.3 *p* > 0.001 Figure 4B) and frequency of attacks (*H* = 26.4; *p* > 0.001 Figure 4C). The analysis of attack latency showed that dams displayed faster attacks than godmothers (*p* = 0.001), pup-sensitized (*p* = 0.023) and pup-naïve females (*p* < 0.001). However, there were no significant differences between godmothers, pup-sensitized and pup-naïve females (all *p* > 0.1). Concerning attack duration, dams spent more time attacking intruders and a higher frequency of aggressive behaviors as compared to all the non-lactating female groups (all of *p* < 0.005). The pairwise comparisons between godmothers, pup-sensitized and pup-naïve virgins showed that all of them behaved in a similar way, with low level of aggressiveness (*p* > 0.1 in all cases).

### Experiment 3. Godmothers do not Display Maternal-Like Aggression Irrespective of the Presence of Pups, but they Express Socio-Sexual Investigation of Males

Contrary to pup-sensitized or pup-naïve virgin females, godmothers have a nest to defend in their home cage. Even so, our previous experiments indicate that in absence of pups, they do not attack male intruders. The next experiment was aimed at checking whether they might show maternal aggression in the presence of pups. We applied a Wilcoxon test to analyze the aggressive behaviors in the same group of godmothers in two experimental conditions, namely in absence and presence of pups. In the latter situation, we observed one infanticide event by a male intruder, and that test was finished at that point.

**Figure 5:**
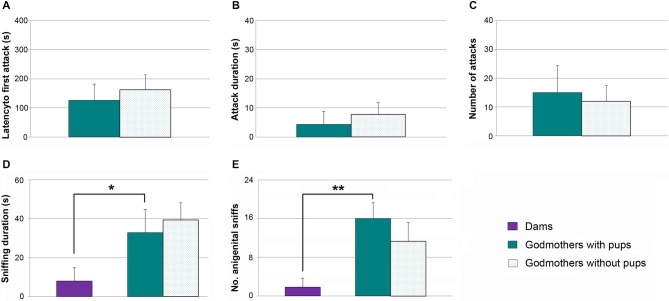
**Godmothers are not aggressive irrespective of the presence of pups, and display more socio-sexual investigation of intruders. (A)** Time (mean ± SEM) that godmothers took to initiate the first attack, **(B)** attack duration and **(C)** number of attacks toward male intruders was low in godmothers, and was not altered in the presence of the pups. The socio-sexual interactions with the male were higher in godmothers than in dams. **(D)** Time spent sniffing the intruder, **(E)** frequency of anogenital approaches. *p < 0.05; **p < 0.01.

Data revealed that there were no statistically significant differences between both tests. Thus, godmothers behaved in a similar way in absence and presence of pups, showing low levels of aggression in all the measures (latency to first attack, Figure 5A), total time duration of attacks (Figure 5B) and frequency of attacks (Figure 5C, *p* > 0.4 in all of cases).

Since godmothers did not attack males, we checked whether there were social (i.e., sniffing) or sexual (i.e., anogenital approaches, lordosis) interactions with them. A Wilcoxon test revealed that time that godmothers spent sniffing the males was not different in the two experimental conditions (with and without pups, *p* = 0.463). Further, a Mann-Whitney test between godmothers and a group of dams in the without pups condition revealed that godmothers sniffed at intruder males significantly more than dams (*p* = 0.026; Figure 5D).

The number of times that godmothers investigated the anogenital region of the males was not significantly different in the presence and the absence of pups (Wilcoxon test, *p* = 0.273; Figure 5E). In addition, godmothers displayed a higher number of anogenital approaches to intruder males as compared to dams (Mann-Whitney test, *p* = 0.004; Figure 5E). Finally, we did not observe any lordosis. Thus, godmothers did not attack males irrespective of the presence of pups, but displayed more socio-sexual interaction with males than dams.

### Experiment 4. Dams do not Display Maternal Aggression in a Neutral Arena

**Figure 6:**
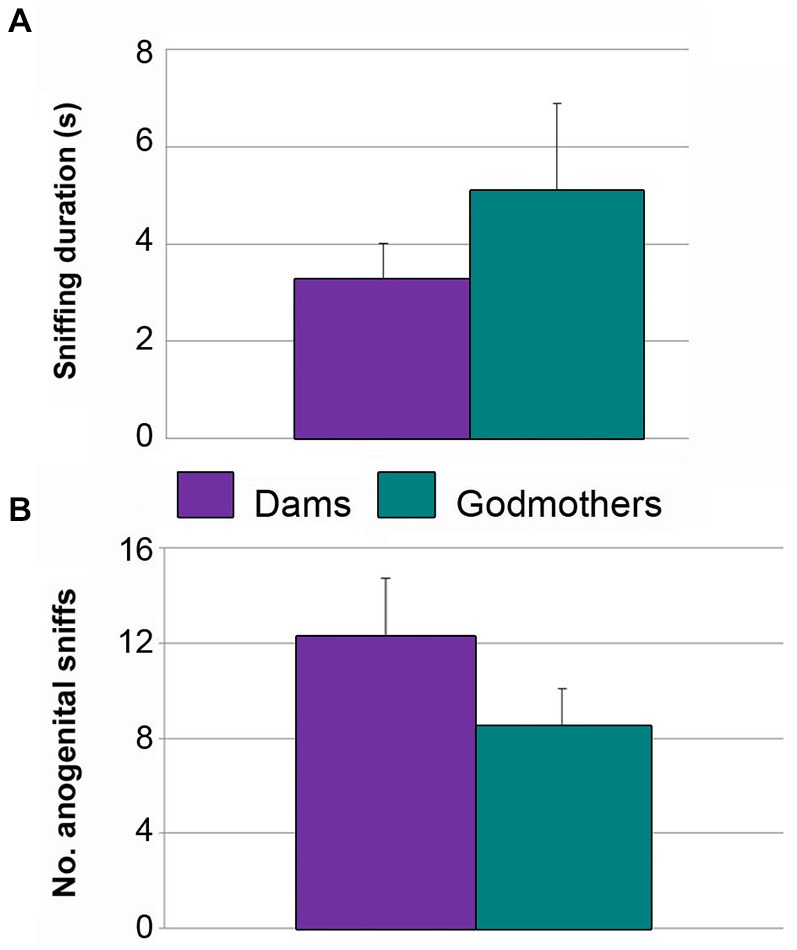
**Dams and godmothers did not differ in socio-sexual investigation of a stimulus conspecific in a neutral arena. (A)** Time (mean ± SEM) that dams and godmothers spent sniffing the intruder. **(B)** Number of anogenital approaches of dams and godmothers to intruders.

Dams and godmothers did not attack males in the neutral arena. Further, they did not differ in the total time sniffing the males (*p* = 0.684; Figure 6A) or anogenital approaches (*p* = 0.280; Figure 6B). These data suggest that maternal aggression is a territorial behavior.

## Discussion

In this study, we have characterized two models of maternal sensitization in virgin female laboratory mice of the outbred strain CD1. In one model, the godmother, virgin females share pup care with dams from parturition, whereas in the second model, virgin females were exposed 2 h daily to pups. In both models, we have studied the expression of the two main components of maternal behavior, namely pup-directed (Rosenblatt, 1967; Numan and Insel, 2003) and non-pup-directed behaviors, i.e., maternal aggression (Numan and Insel, 2003). We extend previous findings showing that contact with foster pups is able to almost spontaneously induce the former but not the latter kind of maternal behavior in virgin females.

### Exposure to Pups Elicits Pup-Directed Maternal Behavior in Virgin Female Mice

We compared pup-oriented behaviors induced by the two different protocols of exposure to foster pups in non-lactating females with the full maternal behavior expressed by lactating dams. Both protocols of maternal sensitization successfully induced pup-directed behaviors with different time course, so that godmothers were indistinguishable from dams in most behavioral aspects from the first testing day (on PPD 2), whereas pup-sensitized females took at least two sensitization sessions to express similar levels of maternal-like behavior. This result is consistent with a previous study (Stolzenberg and Rissman, 2011).

Godmothers, exposed to pups from the moment of parturition, were as quick as dams in the pup-retrieval test. By contrast, pup-sensitized females were slower than dams during both the first day of testing—when they had contact with pups for the first time—and the second. Across tests, pup-sensitized females got experience with pups and they learned to retrieve them faster. Thus, during the third test, after 2 days of 2 h exposure to pups, pup-sensitized females showed the same speed than dams in pup retrieval.

This quick induction of maternal care observed in adult virgin mice contrasts with the situation in virgin adult rats, which need a full week of sensitization to pups to express maternal-like behavior (Fleming and Luebke, 1981). In fact, virgin adult female rats express neophobic avoidance responses when they are presented for the first time with foster young. These responses last for 2–3 days, and after 7 days of exposure, virgin female rats express maternal care. Thus, it is possible that the interspecies difference is due to a reduced neophobic response in mice as compared to rats. Actually, we did not find differences in the latency to approach and sniff the pups between groups. This indicates that virgin females with no previous experience with pups show no aversion for them, as they approach pups with a similar latency than dams. On the other hand, stimuli eliciting fear on anxiety elicit risk assessment behavior in rodents. Our findings show an extremely low level of risk assessment episodes toward pups, which are similar in dams and both groups of virgin females. This indicates that, in contrast to rats, adult CD1 female mice do not display avoidance responses or fear/anxiety toward pups.

Regarding pup licking/grooming, pup-sensitized females overexpress this behavior as compared to dams and godmothers. We hypothesize that these high levels of licking/grooming might correlate with an increased investigative behavior induced by novelty (Rinaldi et al., 2010). Thus, dams and godmothers recognize their young or familiar pups, respectively and, after retrieving them in the nest, express low levels of pup licking/grooming. By contrast, virgin females in the process of sensitization show a higher level of licking/grooming, likely because pups are novel stimuli for them. This hypothesis is in agreement with a previous study by Stolzenberg and Rissman (2011), in which they observed that experienced females were faster in pup retrieval but expressed lower frequency of licking/grooming than females that had never had previous contact with pups. In addition, Alsina-Llanes et al. (2015) also showed that more experienced female mice spend less time both licking pups and in the nest during the pup-retrieval tests. This further supports that pups are neither aversive, nor fear eliciting but, on the contrary, a highly attractive stimulus for previously pup-inexperienced virgin females.

The results on pup licking/grooming contrast with those on crouching. Godmothers showed higher frequency of crouching than dams. The relatively short time that dams spent crouching over their pups is somewhat surprising. We speculate that dams might actually spend more time in the nest while nursing undisturbed, but under the experimental conditions in a room different from the homeroom, and in the absence of any threat, they might be more willing to leave the nest and explore the surroundings—this is the most common behavior shown by the dams. Indeed, it has been proposed that anxiety levels are decreased and exploratory activity increased in lactating females (Bridges, 2015), in agreement with this hypothesis. Godmothers, on the contrary, might be more anxious than dams when left alone with pups, so they are highly motivated to retrieve pups and stay with them in the nest.

In summary, we have shown that godmothers are as fast as dams in the pup retrieval test from the first test day and that they overexpress crouching, suggesting that these virgin accompanying females care for foster pups as much as lactating animals do. These results are in agreement with a previous study about communal nesting in laboratory mice by Gandelman et al. (1970), who observed that virgin females accompanying the dams cared for the young even more than lactating females did in about half of the observations, noting that accompanying females could even act as “midwives”—helping dams during delivery and eating the placenta.

Although we have observed that both lactating and non-lactating females care for the youngsters, previous results suggest that the motivation towards them might be higher in dams. Thus, Hauser and Gandelman (1985) performed an operant task in which they presented a pup as a reinforcer for lever-pressing. They observed that lactating female mice pressed a lever at much higher rate than virgin females did, suggesting that pups are more effective reinforcers for lactating mice than for virgins. To investigate whether dams in our study were more motivated to retrieve pups, we measured the time difference between sniffing and retrieving the first pup. Dams readily retrieved pups as soon as they sniffed them, so that the difference between both behaviors tended to 0 in all the tests. However, in both godmothers and pup-sensitized females there was a variable time lapse between the moment of first sniff and retrieval in the first test day. This result supports that the motivation towards pups is higher in lactating than in non-lactating female mice. Thus, hormonal events during pregnancy and lactation might promote changes in motivational responses in lactating females. If so, lactating female mice would differ from virgin females in their brain dopaminergic circuitry and/or in motivation modulatory systems, as we discuss below.

### Maternal Aggression is Only Present in Lactating Females

Contact with pups inducing full pup-directed maternal behavior is not enough to promote maternal aggression, a separate component of the maternal behavioral repertoire. Indeed, in our experiments, neither constant nor 2 h daily exposure to pups, were able to trigger aggressive behavior in virgin female mice. The lack of aggression in godmothers is especially relevant. Maternal aggression only occurs near the nest, as dams do not attack unknown adult males if encounters occur in an environment other than the dam’s home cage (Experiment 4). This clearly indicates that maternal aggression is territorial and represents a defense of the nest. Therefore, the lack of aggression of the pup-sensitized females in Experiment 2 could be attributed to the lack of a stable nest to defend in their home cages. By contrast, our godmothers had a nest to defend but, even so, they did not express maternal-like aggression. This demonstrates that prolonged (5 days), intimate contact with pups is not able, *per se*, to elicit maternal aggression in virgin female mice, and is consistent with previous reports in mice (Martín-Sánchez et al., 2015) and rats (Erskine et al., 1980b). Therefore, in contrast to maternal care, maternal aggression seems to require physiological changes occurring only in the dams, likely related to endocrine agents acting during pregnancy, parturition and/or lactation.

This finding contrasts, however, with the results by McDermott and Gandelman (1979) that showed that some virgin female mice displayed aggression after 9 days of continuous exposure to 1 day-old pups that were renewed every day. Therefore, there is conflicting evidence relative to the factors promoting the onset of maternal aggression. Early studies suggested that sensory cues from the pups were the key factor triggering maternal aggression in mice. In particular suckling-induced nipple stimulation—but not lactation—would trigger and maintain maternal aggression in mouse dams (Svare et al., 1980). This was supported by two main lines of evidence. On the one hand, parturient females thelectomyzed prepartum or immediately postpartum became not aggressive (Svare and Gandelman, 1976a). On the other hand, Svare and Gandelman (1976b) ovariectomized virgin females and treated them with estradiol benzoate and progesterone for 19 days to induce nipple growth, and fostered them with pups that were observed to attach themselves to the nipples. Treated virgin females exhibited aggression, but not milk production.

Therefore, the lack of aggression in our godmothers suggests that endocrine agents rather than pup-derived stimulation are inducing nest defense. This seems contradicted by previous studies indicating a causal role of nipple stimulation, instead of endocrine factors, in maternal aggression onset and maintenance.

However, a key role of endocrine agents in maternal aggression is further supported by the expression of maternal aggression before parturition, in late-pregnant mice (Mann et al., 1984, who called it pregnancy-induced aggression) and rats (Caughey et al., 2011), in which no nipple stimulation by pups has occurred yet. Maybe it is the hormonal stimuli leading to nipple growth, together with the presence of a nest to defend, rather than nipple stimulation *per se*, what causes maternal attacks. This would fit both our results and those from other studies. Thus, in the experiments by Svare and Gandelman (1976b), the hormonal treatment leading to nipple growth rather than subsequent pup suckling might have induced aggression. In the same vein, McDermott and Gandelman (1979) reported that those virgin females exposed to 1-day-old pups for 9 days that displayed maternal aggression showed enlarged and more numerous nipples than their counterparts that, in the same conditions, were not aggressive. This indicates that a continuous exposure to pups for 9 days had caused hormonal changes leading to both nipple growth and aggressiveness, by means of unknown mechanisms. Apparently these hormonal changes did not occur in our godmothers even if they had spent 5 days caring for pups, likely because of the presence in the same cage of the dam nursing the pups for most of the time.

Finally, sensory stimulation might be important for maintaining rather than for triggering maternal aggression. In fact, it has been shown that the display of aggressive behavior becomes independent of circulating hormones after about day 5 of lactation (Erskine et al., 1980a), suggesting an enduring modification of neural pathways controlling aggression during late pregnancy and first postpartum days.

In conclusion, at least in mice, maternal aggression and pup-directed behaviors seem to use different neural and endocrine mechanisms. These results point to the existence of some physiological events during pregnancy, delivery and lactation that elicit increased motivation for pups and stimulate aggressive behavior. Likely, these events induce central changes in neural pathways controlling these behavioral components.

### Mice as Advantageous Models for the Study of the Neurobiology of Maternal Care and Aggression

Our behavioral results in lactating and virgin females and those reviewed above fully support previous ideas based on different lines of evidence (reviewed by Lonstein and Gammie, 2002; Numan and Insel, 2003; Gammie, 2005; and Numan and Woodside, 2010) about the existence of distinct neural pathways and mechanisms for the control of the two main components of maternal behavior, namely pup care and maternal aggression. However, the circuits involved in both components of maternal behavior share at least two important neural centers with differential roles, namely the lateral septum and medial preoptic area plus the ventral part of bed nucleus of stria terminalis (MPOA-vBST).

The MPOA-vBST continuum seems to be the effector structure of maternal care. Thus, lesions of the MPOA-vBST with fiber-sparing neurotoxic drugs dramatically reduce pup retrieval and nursing (Numan and Numan, 1996; Numan et al., 2005), leading to severe pup weight loss. In addition, there is evidence suggesting that this center is also involved in maternal aggression (Gammie and Nelson, 2001; Hasen and Gammie, 2005). These data suggest that MPOA-vBST could be the convergent point where both maternal care and maternal aggression pathways are coordinated.

Within the MPOA-vBST, the nonapeptides oxytocin and vasopressin might participate in the regulation of maternal behaviors, including aggression. In fact, pharmacological blockade of the receptors for both peptides in the MPOA have been shown to impair the onset of maternal care in the rat (Pedersen et al., 1994). In addition, the expression of receptors for oxytocin and vasopressin has been shown to vary across the peripartum period, coinciding with the highest levels of maternal aggression (Caughey et al., 2011).

However, all these cited studies were performed in rats, which, contrary to mice (this work, Stolzenberg and Rissman, 2011), show no spontaneous maternal care but need a long period of sensitization of about a week of contact with pups to start retrieving pups and hovering over them. Therefore, a comparative analysis of the MPOA-vBST regions and of the possible changes in the nonapeptidergic systems in mice and rats would shed light on the neural substrate of maternal care.

An interesting finding of the present study was that maternal aggression occurs only in the home cage, demonstrating that it is a territorial behavior. This suggests that the hippocampal formation might be involved in the regulation of maternal aggression. Future studies testing this possibility would provide grounds to include the hippocampus as a node of the maternal brain.

### Conclusion

In this article, we have characterized pup-directed and non-directed components of maternal behavior in two models of maternal sensitization. We conclude that godmothers represent a good model of maternal sensitization, which present some advantages. First, it is an easy protocol that minimizes handling of the animals, since they are exposed in their home cages to foster pups. This continuous exposure induces the full expression of pup-directed behaviors, mostly indistinguishable from dams at postnatal day 2. By contrast, our protocol failed to induce maternal-like aggression. These results suggest, on the one hand, that the expression of pup-directed behavior is wired in the brain of female mice, and can be almost spontaneously triggered by contact with pups. On the other hand, the expression of maternal aggression needs some additional internal trigger, like the physiological changes taking place during pregnancy and/or lactation. Thus, we propose that the use of godmothers will help understand the modifications induced by the physiological events that take place in the maternal brain and promote the onset of maternal aggression.

## Author Contributions

EL and FM-G designed research; AM-S, GV-M and AH-M performed research; AM-S, GV-M and CA-P analyzed data; AM-S, FM-G and CA-P wrote the paper, EL, FM-G and CA-P revised the final version and approved the manuscript.

## Conflict of Interest Statement

The authors declare that the research was conducted in the absence of any commercial or financial relationships that could be construed as a potential conflict of interest.
