# Corrigendum: Wired for motherhood: induction of maternal care but not maternal aggression in virgin female CD1 mice

**DOI:** 10.3389/fnbeh.2021.671259

**Published:** 2021-04-30

**Authors:** Ana Martín-Sánchez, Guillermo Valera-Marín, Adoración Hernández-Martínez, Enrique Lanuza, Fernando Martínez-García, Carmen Agustín-Pavón

**Affiliations:** ^1^Laboratory of Functional Neuroanatomy (NeuroFun), Unitat Predepartamental de Medicina, Facultat de Ciències de la Salut, Universitat Jaume I, Castelló de la Plana, Spain; ^2^Departaments de Biologia Cel·lular i de Biologia Funcional, Facultat de Ciències Biològiques, Universitat de València, València, Spain

**Keywords:** aggression, communal nesting, maternal care, maternal sensitization, outbred strain

In the original article, there was an error. We were informed by Dr. Bruce S. Svare that our interpretation of their results published in Svare and Gandelman (1976) was incorrect, and hence we hereby request the following correction.

Corrections have been made to *Discussion, Maternal Aggression is Only Present in Lactating Females, Paragraphs 2 and 4*. The corrected paragraphs are shown below.

This finding contrasts, however, with the results by McDermott and Gandelman (1979) that showed that some virgin female mice displayed aggression after 9 days of continuous exposure to 1 day-old pups that were renewed every day. Therefore, there is conflicting evidence relative to the factors promoting the onset of maternal aggression. Early studies suggested that sensory cues from the pups were the key factor triggering maternal aggression in mice. In particular suckling-induced nipple stimulation—but not lactation—would trigger and maintain maternal aggression in mouse dams (Svare et al., 1980). This was supported by two main lines of evidence. On the one hand, parturient females thelectomyzed prepartum or immediately postpartum became not aggressive (Svare and Gandelman, 1976a). On the other, Svare and Gandelman (1976b) ovariectomized virgin females and treated them with oestradiol benzoate and progesterone for 19 days to induce nipple growth, and fostered them with pups that were observed to attach themselves to the nipples. Treated virgin females exhibited aggression, but not milk production. Importantly, hormone-treated virgin females did not display maternal aggression before suckling stimulation from newborn pups, suggesting that hormonal treatment *per se* is not able to provoke aggressive behavior (Svare and Gandelman, 1976b).

(…)

However, a key role of endocrine agents in maternal aggression is further supported by the expression of maternal aggression before parturition, in late-pregnant mice (Mann et al., 1984, who called it pregnancy-induced aggression) and rats (Caughey et al., 2011), in which no nipple stimulation by pups has occurred yet. Maybe it is the hormonal stimuli leading to nipple growth, together with the presence of a nest to defend, rather than nipple stimulation *per se*, what causes maternal attacks. This would fit both our results and those from other studies. McDermott and Gandelman (1979) reported that those virgin females exposed to 1-day-old pups for 9 days that displayed maternal aggression showed enlarged and more numerous nipples than their counterparts that, in the same conditions, were not aggressive. This indicates that a continuous exposure to pups for 9 days had caused hormonal changes leading to both nipple growth and aggressiveness, by means of unknown mechanisms. Apparently these hormonal changes did not occur in our godmothers even if they had spent 5 days caring for pups, likely because of the presence in the same cage of the dam nursing the pups for most of the time.

The authors apologize for this error and state that this does not change the scientific conclusions of the article in any way. The original article has been updated.
